# The Prevalence and Genetic Diversity of Porcine Circoviruses (PCVs) in Eastern China During 2010–2016 and 2023–2024

**DOI:** 10.3390/vetsci13070657

**Published:** 2026-07-07

**Authors:** Mingyue Wan, Weizhen Shen, Peng Wang, Mengran Zhang, Jing Chen, Bin Zhou

**Affiliations:** 1 MOE Joint International Research Laboratory of Animal Health and Food Safety, College of Veterinary Medicine, Nanjing Agricultural University, Nanjing 210095, China; evelynwan24@126.com (M.W.); 15057302027@163.com (W.S.); meng_ran_zhang@163.com (M.Z.); t2025065@njau.edu.cn (J.C.); 2 Key Laboratory of Animal Bacteriology, Ministry of Agriculture and Rural Affairs, Nanjing Agricultural University, Nanjing 210095, China; 3 Rural Work Management Service Center, Nanjing Jiangbei New Area, Nanjing 210031, China; wangshui9@163.com; 4 College of Veterinary Medicine, Northeast Agricultural University, 600 Changjiang Street Xiangfang District, Harbin 150030, China; 5 Key Laboratory of Diagnosis and Treatment for Epizootic Diseases of Animals in Cold Regions of Heilongjiang Province, Northeast Agricultural University, 600 Changjiang Street Xiangfang District, Harbin 150030, China

**Keywords:** porcine circovirus, PCV2, PCV3, genotype, prevalence, phylogenetic analysis

## Abstract

Although commercial vaccines against porcine circovirus type 2 (PCV2) have been widely implemented globally, PCV2 remains endemic in swine populations, accompanied by ongoing genotype replacement. Meanwhile, the emergence of novel porcine circoviruses (PCVs), including PCV3 and PCV4, has further complicated the prevention and control of porcine circovirus-associated diseases (PCVAD). This study systematically characterized the epidemiological patterns and genetic diversity of PCVs circulating in Eastern China. A total of 739 clinical samples collected between 2010 and 2016 were screened for PCV2. Additionally, 653 samples obtained during 2023–2024 were analyzed using a triplex real-time quantitative PCR (qPCR) assay for the simultaneous detection of PCV2, PCV3, and PCV4. Full-genome amplification and sequencing were subsequently performed on all PCR-positive samples. Epidemiological analysis revealed an overall PCV2 positivity rate of 37.62% during 2010–2016. In the 2023–2024 cohort, the positivity rates for PCV2 and PCV3 were 35.99% and 16.39%, respectively, with a co-infection rate of 10.26%. Notably, no PCV4-positive samples were detected. Phylogenetic analysis demonstrated that PCV2d is the predominant genotype in Eastern China. Furthermore, PCV2g strains were identified in clinical samples for the first time in mainland China, while PCV3b were determined to be the dominant circulating subtype of PCV3. Multiple critical amino acid substitutions were identified within the neutralizing epitopes of the PCV2 Cap protein, and a recombination event involving a PCV2d vaccine strain and a PCV2c reference strain was detected. In contrast, the PCV3 genome exhibited a high degree of genetic conservation. Collectively, these findings expand the molecular epidemiological landscape of PCVs in Eastern China and elucidate the evolutionary dynamics of circulating PCV strains, providing important insights for the development of next-generation vaccines and region-specific PCVAD control strategies.

## Simple Summary

Porcine circoviruses (PCVs) are significant pathogens responsible for substantial economic losses in the global swine industry. Despite the availability of PCV2 vaccines, the virus continues to circulate in pig populations, and emerging circoviruses, including PCV3 and PCV4, have been reported. This study investigated the prevalence and genetic diversity of PCVs in eastern China using samples collected during two distinct periods, 2010–2016 and 2023–2024. The results indicated that PCV2 remained highly prevalent, with PCV2d emerging as the dominant genotype. PCV3 was detected in a considerable proportion of samples, whereas PCV4 was not identified. Notably, the rare PCV2g genotype was identified in clinical samples from China for the first time. Genetic variations potentially associated with immune evasion from current PCV2 vaccines were also observed. These findings provide valuable insights for the optimization of vaccination strategies and the implementation of more effective control measures against PCV infections.

## 1. Introduction

Porcine circovirus (PCV) is a non-enveloped, circular, single-stranded DNA virus belonging to the family *Circoviridae* [1]. To date, four principal genotypes (PCV1-PCV4) have been identified. Among these, PCV1 is considered non-pathogenic, whereas PCV2, PCV3, and PCV4 are recognized as clinically significant pathogens posing substantial threats to the global swine industry. PCV2, first identified in Canada in the 1990s [2], is the etiological agent of postweaning multisystemic wasting syndrome (PMWS) and porcine dermatitis and nephropathy syndrome (PDNS). It frequently co-infects swine with other pathogens, including porcine reproductive and respiratory syndrome virus (PRRSV) and *Haemophilus parasuis* [3,4], resulting in growth retardation in piglets and reproductive failure in sows. Collectively, these infections constitute a major contributor to significant economic losses in intensive pig production systems worldwide. PCV3 was initially identified in the United States in 2015 [5] through metagenomic next-generation sequencing (mNGS) and has subsequently been reported in all major swine-producing regions. Retrospective epidemiological investigations have demonstrated that PCV3 has been circulating covertly in European swine populations since the 1990s [6], with clinical manifestations primarily associated with reproductive disorders in sows and multisystemic inflammatory lesions in piglets. In contrast, PCV4 [7], a recently described circovirus, has thus far been sporadically detected in certain regions of Asia [8,9], and its pathogenic potential as well as transmission dynamics remain to be comprehensively elucidated. From an evolutionary perspective, PCV2 exhibits one of the highest mutation rates among DNA viruses, characterized by rapid genotype turnover driven by elevated spontaneous mutation frequencies and homologous recombination. Conversely, the PCV3 genome displays a high degree of genetic conservation [10], resulting in markedly divergent evolutionary trajectories between these viruses, which continue to be a focal point in veterinary virology research.

Decades of extensive global vaccination have profoundly reshaped the epidemiological landscape of PCV2. Nevertheless, surveillance data from multiple regions indicate that current vaccines are insufficient to eliminate circulating PCV2 from swine populations. Epidemiological reports from Europe and the Americas demonstrate that field PCV2 positivity rates consistently range from 28% to 33% in countries such as Spain and Poland, whereas large-scale commercial farms in South America, including Brazil and Uruguay, report rates as high as 39.2%. In Southeast Asian swine-producing countries, including Vietnam and Thailand, detection rates fluctuate between 33% and 49%, with persistent subclinical circulation of wild-type strains even under comprehensive vaccination programs. From a genotypic standpoint, three successive global shifts in dominant PCV2 subtypes have been documented: PCV2a predominated prior to 2003, followed by replacement by PCV2b between 2004 and 2010, and PCV2d has emerged as the dominant global lineage since 2012. It is widely accepted that sustained immune selective pressure exerted by the extensive use of PCV2a-based vaccines has been a primary driver of these genotype replacement events. In addition to the major genotypes, rare variants ranging from PCV2e to PCV2h have been sporadically identified in Europe and Asia [11,12,13]. Notably, PCV2g has been reported only in a limited number of countries, with scarce epidemiological data available [14,15]. Since its initial identification, PCV3 has disseminated across all major swine-producing regions in North America, Europe, South America, and East Asia [16,17,18,19]. Sero-epidemiological investigations in the United States and Korea [20,21] indicate that PCV3 seropositivity rates in commercial swine herds range from 10% to 18%, while domestic pig populations in Italy and Portugal exhibit approximately 11.2% positivity, and infection rates in wild boar populations exceed 55% [22,23]. Co-infection with PCV2 and PCV3 is widely prevalent in swine production systems worldwide and has been shown to synergistically exacerbate clinical manifestations and pathological lesions [24]. For molecular classification of PCV3, two principal approaches are commonly employed: analysis of key amino acid substitutions (A24V and R27K) within the capsid (Cap) protein coding region [25], or whole-genome sequence analysis (ORF1 + ORF2) [10]. Although geographic clustering of PCV3 subtypes has been observed across different regions, most countries lack longitudinal surveillance data spanning multiple years to comprehensively delineate subtype evolutionary dynamics. To date, PCV4 has been detected only sporadically in a limited number of swine farms in Central and Southeastern Asia and Europe [9,26,27], and its endemic distribution remains to be clearly defined.

China harbors the largest swine population worldwide, with Eastern China functioning as a critical hub for breeding stock introduction and cross-regional live pig movement. Intensive animal trade markedly facilitates the interprovincial dissemination, genetic recombination, and evolutionary diversification of circoviruses. Based on more than a decade of longitudinal surveillance data, this study systematically delineates the spatiotemporal epidemiological patterns and genotypic evolutionary dynamics of PCV2 and PCV3 in Eastern China. The findings augment the foundational molecular epidemiological dataset of porcine circoviruses in this region, enrich East Asian representation within the global PCV evolutionary framework, and provide robust empirical evidence to support the development of next-generation vaccines and the implementation of targeted, precision-based prevention and control strategies for porcine circovirus-associated diseases.

## 2. Materials and Methods

### 2.1. Samples

**Figure 1:**
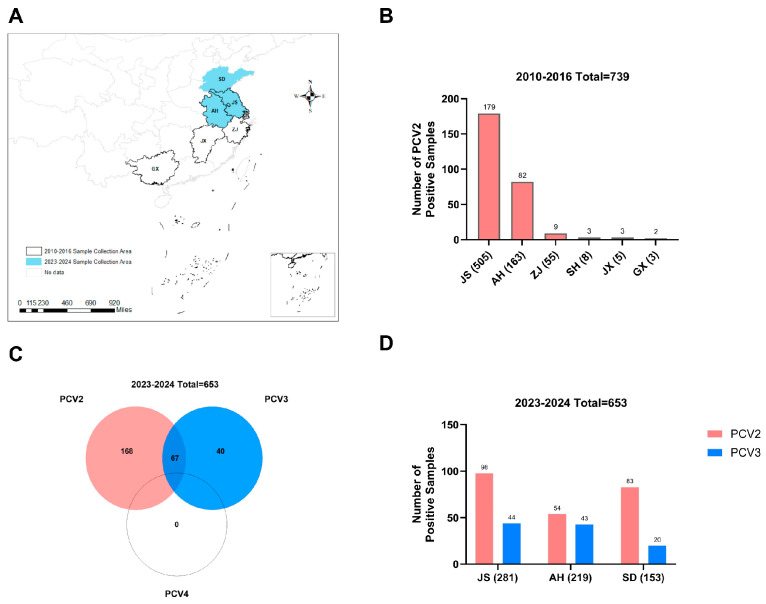
Geographic distribution of samples and PCVs infection status. (**A**) Geographic distribution of collected samples. Black outlines denote provinces sampled during 2010–2016; blue shading denotes provinces sampled during 2023–2024. (**B**) Number of PCV2-positive samples in each province during 2010–2016. The total number of samples tested per province is indicated in parentheses on the x-axis. Detailed positivity rates and sample counts are provided in Table S1. (**C**) Venn diagram showing PCVs infection patterns during 2023–2024. (**D**) Number of PCV2- and PCV3-positive samples in each province during 2023–2024. The total number of samples tested per province is indicated in parentheses on the x-axis. Detailed positivity rates and sample counts are provided in Table S2.

During 2010–2012 and 2015–2016, a total of 739 field samples (lung and lymph node tissues) were collected from pig farms in six regions of southeastern China, including Jiangsu, Anhui, Zhejiang, Jiangxi, Guangxi, and Shanghai (Figure 1A). The pigs were suspected to have clinical symptoms of PMWS and/or PDNS.

Between 2023 and 2024, an additional 653 samples were obtained from pig farms and slaughterhouses across three provinces in eastern China (Jiangsu, Anhui, and Shandong) (Figure 1A). Among these, 518 samples consisted of lymph node tissues collected from apparently healthy pigs at slaughterhouses, while the remaining 135 field samples obtained from pigs with clinical symptoms suggestive of porcine circovirus disease (PCVD) on different farms, including lung, kidney, heart, blood, and testicular fluid.

### 2.2. Viral DNA Extraction and Virus Detection

Tissue samples (approximately 200 mg each) were finely minced and homogenized in a five-fold volume of phosphate-buffered saline (PBS). The homogenates were subjected to three freeze–thaw cycles to facilitate viral particle release, followed by centrifugation at 12,000× *g* for 5 min at 4 °C. Subsequently, 200 μL of the supernatant was collected for viral DNA extraction using DNAzol^®^ Reagent (Invitrogen, Carlsbad, CA, USA) in accordance with the manufacturer’s instructions.

All samples collected during 2010–2016 were screened for PCV2 using a real-time fluorescent quantitative PCR assay based on the Chinese national standard (GB/T 34745-2017) [28]. Samples collected during 2023–2024 were analyzed using a previously established triplex real-time qPCR assay developed in our laboratory [29], enabling the simultaneous detection of PCV2, PCV3, and PCV4.

### 2.3. PCV2 and PCV3 Whole-Genome Sequencing

To obtain full-length genomic sequences of PCV2 and PCV3 for subsequent phylogenetic analysis, specific primers (Table 1) were employed for gene amplification. The PCR reaction mixture comprised 25 μL of 2 × Rapid Taq Master Mix (Vazyme, Nanjing, China), 2 μL each of forward and reverse primers (10 μM), 2 μL of template DNA, and sterile ddH_2_O to a final volume of 50 μL. The amplification conditions were as follows: initial denaturation at 95 °C for 3 min; 35 cycles of denaturation at 98 °C for 15 s, annealing at 60 °C for 30 s, and extension at 72 °C for 15 s; and a final extension at 72 °C for 5 min. PCR products were analyzed by 1% agarose gel electrophoresis, purified, and subsequently cloned into the pMD18-T vector (TaKaRa, Beijing, China), followed by transformation into DH5α competent cells. Positive clones, verified by PCR using M13 primers, were subjected to Sanger sequencing at a commercial facility (Sangon Biotech, Shanghai, China).

### 2.4. Bioinformatic Analysis

The complete genome sequences of PCV2 and PCV3 were edited and assembled using the EditSeq program (DNA STAR, Madison, WI, USA) [30]. Reference strains and genomic sequences obtained in this study are summarized in Supplementary Tables S4 and S5. Multiple sequence alignments were conducted using Molecular Evolutionary Genetics Analysis (MEGA) software version 11.0.13. Phylogenetic trees were constructed using the Neighbor-Joining (NJ) method with 1000 bootstrap replicates implemented in MEGA [10,31]. Mutational analyses of the Cap protein were performed using BioEdit v7.0.9.0. Potential recombination events were further evaluated using RDP4 and Simplot v3.5.1 software [32,33].

## 3. Results

### 3.1. Prevalence and Geographical Distribution of PCV2 and PCV3

To assess the epidemiological prevalence of PCV2, a systematic survey was conducted in southeastern China based on field samples collected during 2010–2012 and 2015–2016. Viral DNA was extracted from a total of 739 samples and subsequently analyzed using a standardized qPCR assay (GB/T 34745-2017) [28]. The overall PCV2 positivity rate was 37.62% (278/739). Notably, the positivity rates in Anhui and Jiangsu provinces were 50.31% (82/163) and 35.45% (179/505), respectively (Figure 1B, Table S1). Due to limited sample sizes from Zhejiang, Shanghai, Jiangxi, and Guangxi, only sporadic PCV2 detection was observed in these regions, underscoring the necessity for enhanced epidemiological surveillance. Collectively, PCV2 infection was widely distributed across southeastern China. Temporal analysis revealed that the highest positivity rate occurred in 2011 (54.17%, 65/120), whereas the lowest was recorded in 2012 (29.35%, 54/184). Positivity rates in the remaining years consistently exceeded 30% (Table S3), indicating sustained and high-level circulation of PCV2 throughout the study period.

With the emergence of novel porcine circoviruses, a comprehensive epidemiological investigation encompassing PCV2, PCV3, and PCV4 was conducted in eastern China in 2025 (Figure 1A). A total of 653 samples collected during 2023–2024 were analyzed. The overall positivity rates of PCV2 and PCV3 were 35.99% (235/653) and 16.39% (107/653), respectively (Table S2), with a co-infection rate of 10.26% (67/653) (Figure 1C). Regionally, the PCV2 positivity rate in Shandong Province reached 54.25% (83/153), exceeding those observed in Jiangsu (34.86%, 98/281) and Anhui (24.66%, 54/219) (Table S2, Figure 1D). Temporal analysis further indicated PCV2 positivity rates of 47.48% (113/238) in 2023 and 30.84% (128/415) in 2024 (Table S3), reflecting persistently elevated epidemic levels. In contrast, PCV3 detection rates were consistently lower than those of PCV2 across all regions. Provincial PCV3 positivity rates were 13.07% (20/153) in Shandong, 15.66% (43/219) in Anhui, and 19.63% (43/281) in Jiangsu (Table S2, Figure 1D). Notably, no PCV4-positive samples were identified in this study.

A total of 99 complete PCV2 genomes and 10 complete PCV3 genomes were successfully obtained and deposited in the NCBI GenBank database. The corresponding accession numbers are provided in Table S4.

### 3.2. Genetic Analysis of PCV2

**Figure 2:**
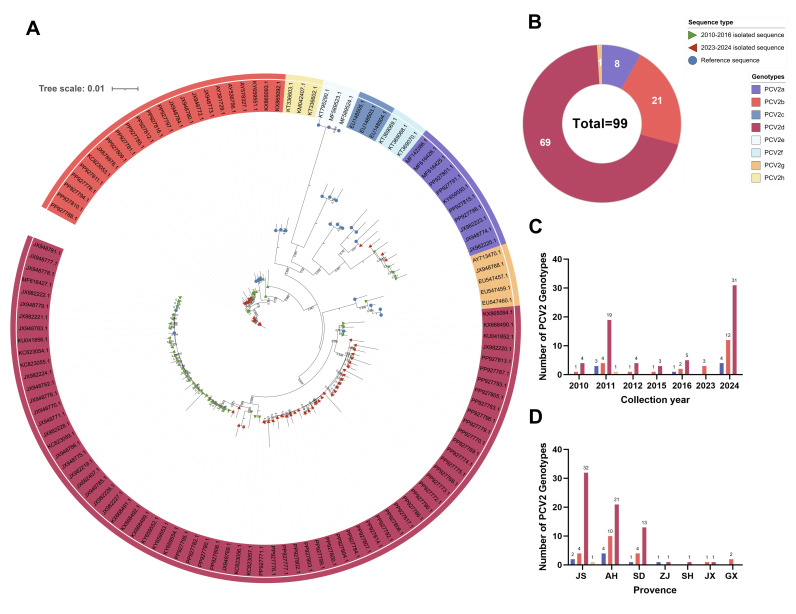
Phylogenetic analysis of PCV2 based on complete genome sequences. (**A**) Phylogenetic analysis of 99 PCV2 isolates and 25 reference strains. The phylogenetic tree was constructed using the Neighbor-Joining (NJ) method with 1000 bootstrap replicates in MEGA 11 software after sequence alignment with Clustal W. (**B**) Genotypic distribution of various PCV2 isolates. (**C**) Genotypic distribution of PCV2 isolates by sampling year. (**D**) Genotypic distribution of PCV2 isolates by province.

A total of 99 complete PCV2 genomic sequences were successfully obtained from 519 PCV2-positive samples, comprising 49 sequences from 2010–2016 and 50 sequences from 2023–2024. These sequences were subjected to phylogenetic analysis in conjunction with 31 reference PCV2 strains retrieved from the GenBank database (Table S5). Genotypic classification revealed that the predominant genotypes PCV2a, PCV2b, and PCV2d accounted for 8.08% (8/99), 21.21% (21/99), and 69.70% (69/99) of the sequenced isolates, respectively (Figure 2A,B). Temporal analysis demonstrated that PCV2d was the predominant genotype across all surveyed years, with the exception of 2023 (Figure 2C), indicating a pronounced evolutionary transition in eastern China from PCV2a (dominant prior to 2010) to the currently prevailing PCV2d lineage. Geographically, PCV2d was the predominant genotype in the three provinces (Jiangsu, Anhui, and Shandong) with sufficient sample sizes (Figure 2D). Notably, one isolate (strain JX948768.1) obtained from Jiangsu Province in 2011 clustered within the PCV2g clade (Figure 2A), representing the first molecular detection and genomic characterization of PCV2g from clinical samples in China. Sequence homology analysis indicated that the 99 PCV2 isolates shared 90.8–99.7% whole-genome nucleotide identity among themselves and exhibited 90.8–99.9% identity with the 31 reference PCV2 strains (Figure S1).

### 3.3. Genetic Analysis of PCV3

**Figure 3:**
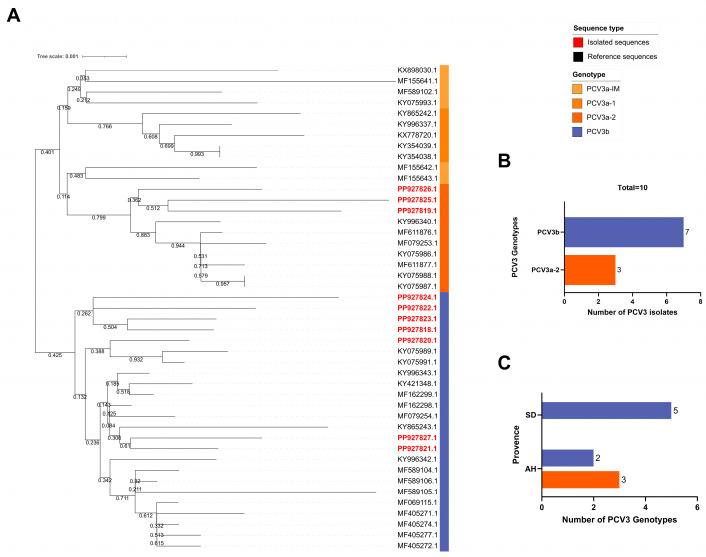
Phylogenetic analysis of PCV3 based on complete genome sequences. (**A**) Phylogenetic analysis of 10 PCV3 isolates and 35 reference strains. The phylogenetic tree was constructed using the Neighbor-Joining (NJ) method with 1000 bootstrap replicates in MEGA 11 software after sequence alignment with Clustal W. (**B**) Genotypic distribution of various PCV3 isolates. (**C**) Genotypic distribution of PCV3 isolates by province.

In this study, 10 complete PCV3 genomic sequences were successfully obtained from 107 PCV3-positive samples. Phylogenetic analysis was performed in comparison with 35 reference PCV3 sequences (Table S5) retrieved from the GenBank database, following established genotyping criteria. The phylogenetic results classified all PCV3 isolates into two major genotypes, PCV3a and PCV3b. The PCV3a genotype was further subdivided into three subclades: PCV3a-IM, PCV3a-1, and PCV3a-2. Among the 10 isolates, three strains were assigned to the PCV3a-2 subclade, whereas the remaining seven strains clustered within the PCV3b lineage (Figure 3A,B). Although PCV3b represented the predominant genotype overall, geographic analysis indicated that PCV3a sequences outnumbered PCV3b sequences in Anhui Province (Figure 3C). Genomic homology analysis demonstrated that the 10 PCV3 isolates shared 98.6–99.6% whole-genome nucleotide identity among themselves and exhibited 98.8–99.7% identity relative to the 35 reference PCV3 strains. These results highlight the high degree of genetic conservation characteristic of PCV3 (Figure S2).

### 3.4. Amino Acid Analysis of Cap Protein

**Figure 4:**
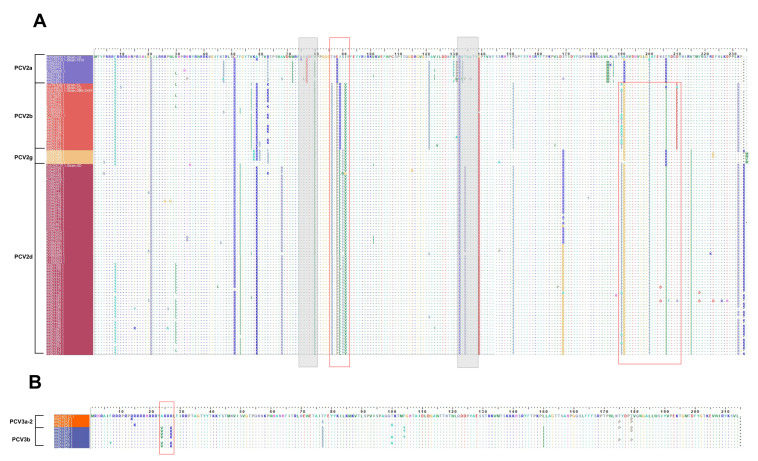
Alignment of capsid protein sequences of PCV2 and PCV3 isolates. (**A**) Alignment of Cap protein sequences of 99 PCV2 isolates. The red solid line box shows the motifs of the PCV2 genotype; the gray highlighted area represents the location of the neutralizing epitope. The asterisk (*) denotes the stop codon. (**B**) Alignment of capsid protein sequences of the 10 PCV3 isolates; the red solid line box shows the amino acid site used for genotyping. The asterisk (*) denotes the stop codon.

The Cap protein encoded by the ORF2 gene represents the sole structural protein of both PCV2 and PCV3 and plays a critical role in viral replication and host immune interactions. In this study, amino acid sequence alignment of the Cap protein was conducted using 99 PCV2 field isolates, vaccine strains representing the three predominant PCV2 genotypes (PCV2a, PCV2b, and PCV2d), and a PCV2g reference sequence. Amino acid variations were primarily concentrated within three hypervariable regions, corresponding to residues 53–91, 121–134, and 185–215. Further characterization of conserved motifs and genotype-specific substitutions within the Cap region revealed that PCV2a strains harbored the canonical motif 86TNKISI91, whereas PCV2b strains exhibited the characteristic 86SNPLTV91 and A/TGIE motifs. Most PCV2d and PCV2g strains displayed 86SNPLTV91 and TGID motifs. However, a subset of PCV2d isolates exhibited distinct variations within these signature motifs, including 86SNPLMG91, 86SNPPTV91, 86SNPLSV91, and TGIN. Additionally, several critical amino acid substitutions were identified within previously reported neutralizing epitopes, namely 75NINDFL80 and 134KANALT139 [34,35]. Notably, in certain isolates, the canonical stop codon was substituted by AAA or AAG, encoding an extra lysine (K) residue and resulting in an extended C-terminus of the Cap protein (Figure 4A, Table 2).

To characterize amino acid variations in the Cap protein of PCV3 isolates, sequence alignment was performed using the earliest reported PCV3 strain 29160 (GenBank accession number: KT869077.1) as the reference. A total of eleven amino acid substitution sites were identified within the PCV3 Cap protein, among which A24V and R27K represented the most prominent variations. Genotypic classification based on these two substitutions indicated that four isolates belonged to the PCV3a genotype (24A, 27R), whereas the remaining six isolates were assigned to the PCV3c genotype (24V, 27K). Notably, all sequences previously classified within the PCV3a-2 subclade consistently harbored the characteristic residues 104F and 150L, whereas all PCV3b sequences contained the 150I residue. Overall, the PCV3 Cap protein exhibited a high degree of genetic conservation throughout its evolutionary trajectory (Figure 4B).

### 3.5. Recombination Analyses

**Figure 5:**
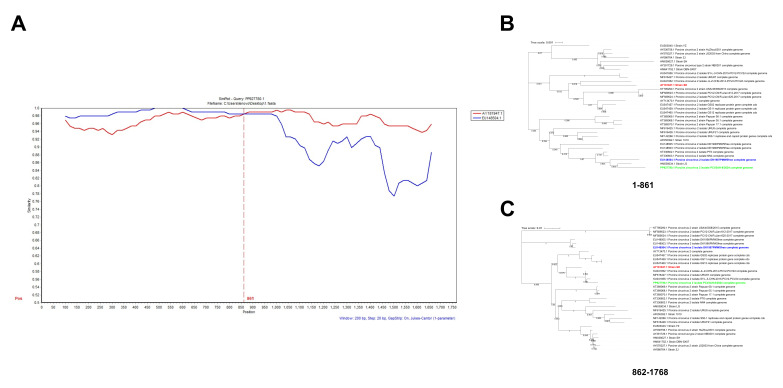
Recombination event of PCV2. (**A**) Simplot recombination analysis of PCV2 AH-8/2024. (**B**,**C**) Phylogenetic trees based on a potential breakpoint located at 861.

Genetic recombination represents a fundamental driver of viral evolution and genetic diversification. In this study, potential recombination events were systematically screened using RDP 4.0 and Simplot software based on the genomic sequences of 99 PCV2 isolates and 31 reference sequences retrieved from the GenBank database. A single credible recombination event (strain PP927780.1) was identified (Figure 5A), arising from recombination between a PCV2d strain (AY181947.1) and a PCV2c strain (EU148504.1). Notably, strain AY181947.1 corresponds to a commercially available vaccine strain widely utilized in China. Simplot analysis further delineated the putative recombination breakpoint at genomic position 861, and the validity of this recombination event was corroborated by phylogenetic reconstruction based on the breakpoint region (Figure 5B,C). In contrast, no recombination signals were detected among the 10 PCV3 isolates obtained in this study.

## 4. Discussion

PCV2 is widely recognized as the primary etiological agent of PCVAD, imposing substantial economic burdens on the global swine industry. Since its initial identification, PCV3 has disseminated extensively worldwide, yet no commercial vaccines are currently available for its prevention and control. Although inactivated and subunit vaccines targeting PCV2 have been extensively developed and implemented, the prevalence of PCV2 infection in China remains persistently high [36]. Previous investigations have reported PCV2 positivity rates exceeding 50% in certain regions of China [37]. In the present study, two independent epidemiological surveys conducted in eastern China during 2010–2016 and 2023–2024 demonstrated that the overall PCV2 positivity rate was 37.62% and remained relatively stable at 35.99% in the later period. These findings indicate that PCV2 has maintained sustained and high-level circulation in eastern China despite decades of widespread vaccination. Consistent with previous reports from Jiangsu Province (41.42%, 1108/2675, 2014–2021) [38], the positivity rates observed in this study (35.45% and 34.86%) further corroborate the endemic nature of PCV2 in this region. Globally, PCV2 prevalence ranges from 33% to 49% in Southeast Asian countries and approximately 30% in European countries [39], placing the epidemiological data obtained in this study within the typical global range and reinforcing the characterization of PCV2 as a globally endemic pathogen [40,41]. Temporal analysis revealed that the PCV2 positivity rate peaked at 54.17% in 2011 and remained consistently above 30% in subsequent years, suggesting that vaccination effectively mitigates disease severity but does not completely interrupt viral transmission within swine populations. Spatially, the markedly higher positivity rate observed in Shandong Province (54.25%) compared to Jiangsu and Anhui may reflect regional disparities in breeding density, animal movement, and farm-level biosecurity practices. Due to limited sample sizes from Zhejiang, Shanghai, Jiangxi, and Guangxi, only sporadic infections were detected in these areas, highlighting the necessity for expanded and continuous surveillance.

As an emerging circovirus, epidemiological data on PCV3 remain relatively limited, with current evidence indicating a substantially lower prevalence compared to PCV2 [42]. In this study, the overall PCV3 positivity rate was 16.39%, with provincial rates ranging from 13.07% to 19.63%. Variability in reported prevalence across regions in China is likely attributable to differences in sampling strategies, temporal frameworks, and detection methodologies. The PCV2/PCV3 co-infection rate reached 10.26%, consistent with previous findings demonstrating that co-infection can synergistically exacerbate respiratory and lymphoid tissue damage, thereby intensifying clinical manifestations [43]. Notably, no PCV4-positive samples were detected, although sporadic cases have been reported elsewhere, underscoring the importance of continued surveillance.

The genetic diversity of PCV2 continues to expand, with the rapid emergence of novel variants. Phylogenetic analysis has classified PCV2 into eight genotypes (a–h) [31], and successive global genotype shifts—from PCV2a to PCV2b and subsequently to PCV2d—have been well documented [44,45,46,47]. In the present study, PCV2d accounted for 69.70% of isolates, followed by PCV2b (21.21%) and PCV2a (8.08%), aligning with prevailing evolutionary trends in China and Southeast Asia. Temporal dynamics further confirmed the progressive replacement of PCV2a by PCV2b and ultimately by PCV2d in eastern China. Although PCV2g sequences have been retrospectively identified in public databases, the lack of detailed epidemiological metadata limits their interpretability [31]. In contrast, the PCV2g strain identified in this study was derived from a well-documented field sample, providing the first direct epidemiological evidence of PCV2g circulation in China and suggesting the ongoing emergence of rare genotypes under immune selective pressure.

The Cap protein, as the sole structural protein of circoviruses, contains major neutralizing epitopes and serves as a critical determinant of host immune recognition. Amino acid substitutions within the Cap protein constitute a key mechanism for immune evasion and host adaptation. In this study, mutations were predominantly localized within three regions (residues 53–91, 121–134, and 185–215), consistent with previously reported antigenic hotspots. Notably, substitutions were identified within the neutralizing epitopes 75NINDFL80 and 134KANALT139, which may contribute to antigenic variation and potentially enhance viral fitness and transmissibility in vaccinated populations. Distinct genotype-specific motifs were observed, with PCV2a harboring 86TNKISI91 and PCV2b/PCV2d predominantly exhibiting 86SNPLTV91. The emergence of novel motif variants (e.g., SNPLMG, SNPPTV) in PCV2d strains further indicates ongoing adaptive evolution under vaccine-induced immune pressure. Additionally, stop codon mutations resulting in lysine extension may alter the structural conformation of the Cap protein, potentially compromising vaccine-induced humoral immunity.

In contrast, PCV3 exhibits high genomic stability and a relatively low mutation rate. The isolates in this study shared over 98.6% genomic identity, consistent with previous reports. Phylogenetic analysis based on complete coding sequences confirmed the classification of PCV3 into two major genotypes, PCV3a and PCV3b [10], with PCV3b predominating overall. However, regional variation was observed, as PCV3a strains were more prevalent in Anhui Province. Classification based on key amino acid substitutions (A24V and R27K) further supported subtype differentiation [25], while characteristic residues (104F/150L and 150I) serve as reliable molecular markers. The high conservation of the PCV3 genome suggests a relatively stable evolutionary trajectory with limited short-term antigenic drift.

Genetic recombination represents a pivotal mechanism driving circovirus diversification and virulence evolution. In this study, a single recombination event was identified in PCV2, involving a widely used PCV2d vaccine strain and a PCV2c reference strain, with a breakpoint at nucleotide position 861. Previous studies have reported similar recombination patterns between PCV2c and PCV2d [48], and vaccine-associated genetic admixture has been implicated as a contributing factor to recombinant emergence [49,50]. Such recombination events may alter viral antigenicity and pathogenicity, thereby posing ongoing challenges to vaccine efficacy and disease control. In contrast, no recombination signals were detected in PCV3 isolates, consistent with its highly conserved genomic architecture.

Vaccination remains a cornerstone for controlling epidemic diseases. Although current PCV2 vaccines effectively reduce disease severity, the continuous emergence of genetically diverse strains necessitates ongoing evaluation of vaccine efficacy. Furthermore, the emergence of novel circoviruses, including PCV3, PCV4, and PCV5, presents additional challenges for disease control. Therefore, it is imperative to systematically evaluate existing vaccines, elucidate the molecular mechanisms underlying PCV pathogenesis, and accelerate the development of next-generation vaccines and precision control strategies.

## 5. Conclusions

The present study substantiates the widespread circulation of PCV2 and PCV3 in Eastern China, with no molecular evidence supporting the presence of PCV4 in local swine populations. Phylogenetic analyses revealed the concurrent circulation of multiple PCV2 genotypes, including PCV2a, PCV2b, and PCV2d. Among these, PCV2d has remained the predominant genotype since 2010, while the PCV2g subtype was identified for the first time in China from clinical samples. Furthermore, PCV3b was determined to be the dominant genotype among circulating PCV3 strains. Amino acid alignment of the Cap protein identified genotype-specific motif variations, substitutions at critical residues within neutralizing epitopes of PCV2, and putative signature amino acid sites distinguishing PCV3 genotypes. In addition, a recombination event involving a vaccine-derived strain was detected. Collectively, these findings provide valuable epidemiological and evolutionary insights into PCV2 and PCV3, thereby establishing a foundational framework for the development of targeted prevention strategies and optimized control measures against porcine circovirus-associated diseases.

## Figures and Tables

**Table 1:** Primers for full-genome amplification of PCV2 and PCV3.

Primer Name	Primer Sequence	Position	Amplified Length
PCV2-F1	5′-ATCCACGGAGGAAGGGGGCCAGTT-3′	920–943	1772
PCV2-R1	5′-ACGTGCTTTCTAGAACAG-3′	900–925	
PCV3-F1	5′-GCTTACCCGGATTTGGTCG-3′	422–440	1291
PCV3-R1	5′-ACAGATGCCAATCAGATCTAGGTAC-3′	1692–1712	
PCV3-F2	5′-GTCGTCTTGGAGCCAAGTG-3′	1616–1634	825
PCV3-R2	5′-CGACCAAATCCGGGTAAGC-3′	422–440	

**Table 2:** Critical amino acid substitutions in the capsid protein of various PCV2 genotypes, i.e., PCV2a, PCV2b, PCV2d, and PCV2g.

Position	PCV2a	PCV2b	PCV2d	PCV2g
8	Y	Y	F(39/69)/Y (30/69)	Y
47	S(7/8)/A(1/8)	T(20/21)/S(1/21)	T	T
53	I	F(19/21)/I(2/21)	I(68/69)/F(1/69)	I
57	V	I	V	V
59	A	R(12/21)/K(9/21)	K	R
63	T(7/8)/S(1/8)	K(12/21)/T(9/21)	R(68/69)/K(1/69)	T
68	A(7/8)/S(1/8)	A	N(67/69)/T(1/69)/A(1/69)	A
77	D	N	N	N
86	T	S	S	S
88	K	P	P	P
89	I	R	L	L
90	S	S	T(67/69)/M(1/69)/S(1/69)	T
91	I	V	V(68/69)/G(1/69)	V
121	S	S	T(67/69)/S (2/69)	T
130	F(7/8)/V(1/8)	V	V	V
131	P(7/8)/M(1/8)	T	T	T
133	S(7/8)/V(1/8)	A	A	A
134	T(7/8)/P(1/8)	T	N(68/69)/T(1/69)	T
151	P	T	T	P
169	S(7/8)/N(1/8)	S	G(40/69)/R(26/69)/T(2/69)/A(1/69)	R
185	M	L	L	L
187	L(7/8)/I(1/8)	I	I	I
190	S	T(13/21)/A(8/21)	T(66/69)/A(3/69)	S
206	K	I	I	K
210	D	E	D(68/69)/N(1/69)	D
215	V	V	I	I
232	K	N	N(67/69)/K(2/69)	N

## Data Availability

The original contributions presented in this study are included in the article/supplementary material. Further inquiries can be directed to the corresponding author.

## Supplementary Materials

The following supporting information can be downloaded at: https://www.mdpi.com/article/10.3390/vetsci13070657/s1. Figure S1: Heatmap illustrating the percentage identity between 99 PCV2 isolate strains and 31 reference strains based on whole-genome sequences; Figure S2: Heatmap illustrating the percentage identity between 10 PCV3 isolates and 35 reference strains based on whole-genome sequences; Table S1: Prevalence of PCV2 in collected samples during 2010–2016 from different provinces; Table S2: Prevalence of PCV2 and PCV3 in collected samples during 2010–2016 from different provinces; Table S3: Prevalence of PCV2 and PCV3 in different years; Table S4: The information on PCV2 and PCV3 strains was obtained in this study..
