# Supplementary material for: The Prevalence and Genetic Diversity of Porcine Circoviruses (PCVs) in Eastern China During 2010–2016 and 2023–2024
Source: Vet Sci. 2026 Jul 7;13(7):657. doi: 10.3390/vetsci13070657 (PMC13418248; doi:10.3390/vetsci13070657)
Supplement: Supplementary file 1 [file vetsci-13-00657-s001.zip › vetsci-4412819-supplementary.pdf]

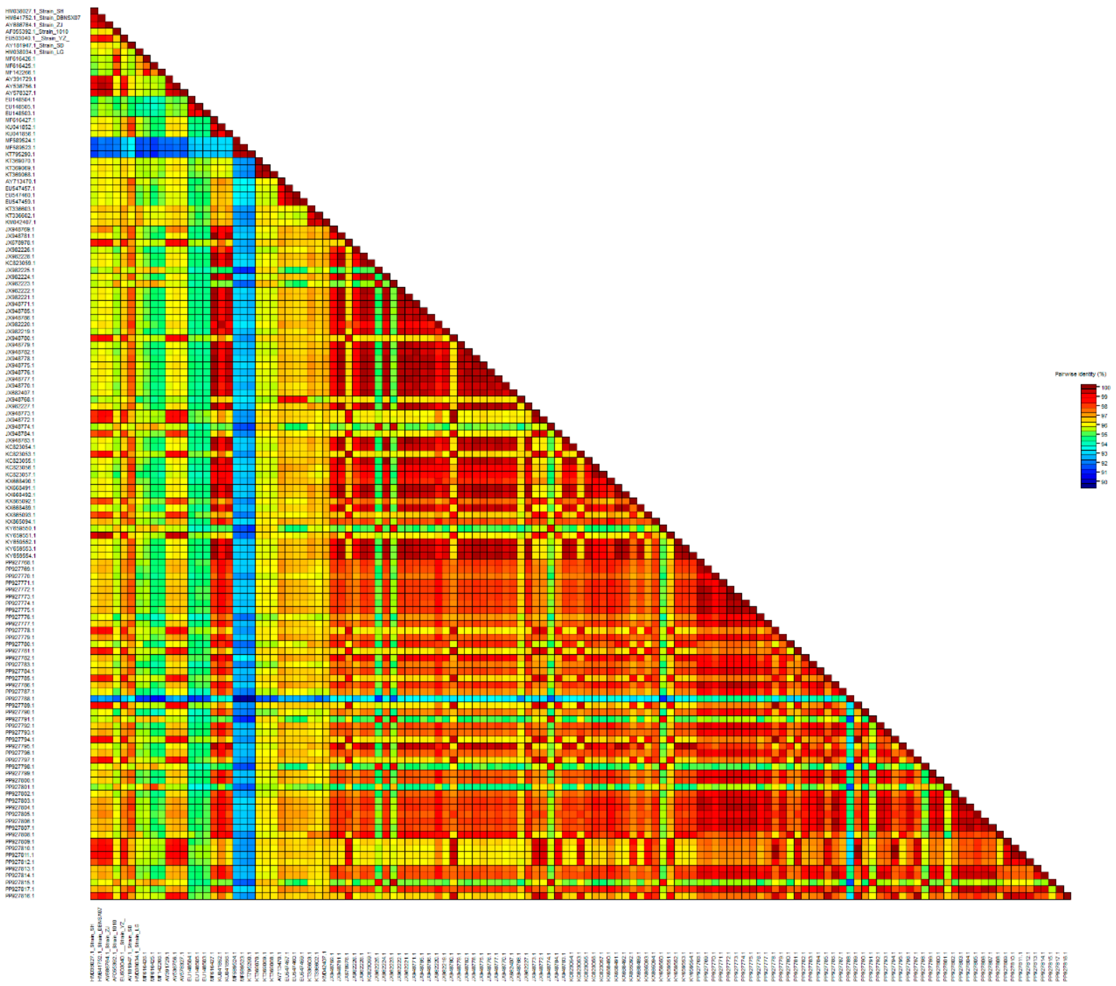

Figure S1. Heatmap illustrating the percentage identity between 99 PCV2 isolate strains and 31 reference strains based on whole-genome sequences.

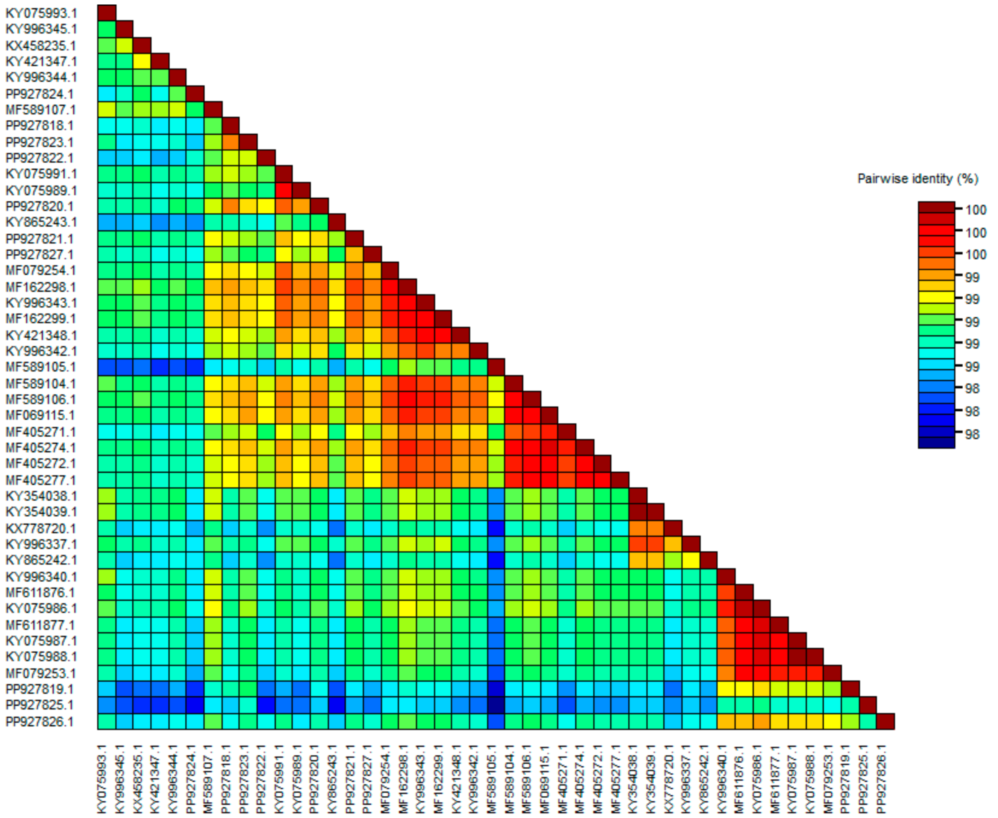

Figure S2. Heatmap illustrating the percentage identity between 10 PCV3 isolates and 35 reference strains based on whole-genome sequences.

Table S1. Prevalence of PCV2 in collected samples during 2010-2016 from different provinces

Provence	Positive samples	Tested samples	Positive Rate (%)
Jiangsu	179	505	35.45%
Anhui	82	163	50.31%
Zhejiang	9	55	16.36%
Shanghai	3	8	37.50%
Jiangxi	3	5	60.00%
Guangxi	2	3	66.67%
Total	278	739	37.62%

Table S2. Prevalence of PCV2 and PCV3 in collected samples during 2010-2016 from different provinces

Provence	Tested samples	PCV2 Positive Rate	PCV3 Positive Rate
Jiangsu	281	98, 34.86%	44, 15.66%
Anhui	219	54, 24.66%	43, 19.63%
Shandong	153	83, 54.25%	20, 13.07%
Total	653	235, 35.99%	107, 16.39%

Table S3. Prevalence of PCV2 and PCV3 in different years.

Year	PCV2 positive samples	PCV3 positive samples	Tested samples	PCV2 Positive Rate	PCV3 Positive Rate
2010	49	/	137	35.77%	/
2011	65	/	120	54.17%	/
2012	54	/	184	29.35%	/
2015	59	/	163	36.20%	/
2016	51	/	135	37.78%	/
2023	107	51	238	47.48%	21.42%
2024	128	56	415	30.84%	13.49%
Total	513	107	1392	36.86%	/

Table S4. The information on PCV2 and PCV3 strains was obtained in this study.

GenBank accession number	date	area	genotype	GenBank accession number	date	area	genotype
JX948769	2010	Anhui	PCV2d	PP927768	2023	Jiangsu	PCV2d
JX948781	2010	Jiangsu	PCV2d	PP927769	2023	Jiangsu	PCV2d
JX678978	2010	Jiangsu	PCV2b	PP927770	2023	Jiangsu	PCV2d
JX982226	2010	Anhui	PCV2d	PP927771	2024	Shandong	PCV2d
JX982228	2010	Jiangsu	PCV2d	PP927772	2024	Jiangsu	PCV2d
KC823059	2011	Jiangsu	PCV2d	PP927773	2024	Jiangsu	PCV2d
JX982225	2011	Zhejiang	PCV2a	PP927774	2024	Jiangsu	PCV2d
JX982224	2011	Jiangsu	PCV2d	PP927775	2024	Jiangsu	PCV2d
JX982223	2011	Jiangsu	PCV2a	PP927776	2024	Shandong	PCV2d
JX982222	2011	Jiangsu	PCV2d	PP927777	2024	Shandong	PCV2d
JX982221	2011	Jiangsu	PCV2d	PP927778	2024	Anhui	PCV2b
JX948771	2011	Shanghai	PCV2d	PP927779	2024	Anhui	PCV2d
JX948785	2011	Zhejiang	PCV2d	PP927780	2024	Anhui	PCV2d
JX948786	2011	Jiangsu	PCV2d	PP927781	2024	Anhui	PCV2b
JX982220	2011	Jiangsu	PCV2d	PP927782	2024	Anhui	PCV2d
JX982219	2011	Jiangsu	PCV2d	PP927783	2024	Anhui	PCV2d
JX948780	2011	Jiangxi	PCV2b	PP927784	2024	Anhui	PCV2d
JX948779	2011	Jiangxi	PCV2d	PP927785	2024	Shandong	PCV2b
JX948782	2011	Jiangsu	PCV2d	PP927786	2024	Shandong	PCV2d
JX948778	2011	Anhui	PCV2d	PP927787	2024	Shandong	PCV2d
JX948775	2011	Jiangsu	PCV2d	PP927788	2024	Anhui	PCV2b
JX948776	2011	Anhui	PCV2d	PP927789	2024	Anhui	PCV2b
JX948777	2011	Anhui	PCV2d	PP927790	2024	Anhui	PCV2d
JX948770	2011	Jiangsu	PCV2d	PP927791	2024	Shandong	PCV2a

JX682407	2011	Jiangsu	PCV2d	PP927792	2024	Shandong	PCV2d
JX948768	2011	Jiangsu	PCV2g	PP927793	2024	Shandong	PCV2d
JX982227	2011	Jiangsu	PCV2d	PP927794	2024	Shandong	PCV2b
JX948773	2011	Guangxi	PCV2b	PP927795	2024	Anhui	PCV2d
JX948772	2011	Guangxi	PCV2b	PP927796	2024	Anhui	PCV2d
JX948774	2011	Anhui	PCV2a	PP927797	2024	Shandong	PCV2b
JX948784	2011	Jiangsu	PCV2b	PP927798	2024	Anhui	PCV2a
JX948783	2011	Jiangsu	PCV2d	PP927799	2024	Shandong	PCV2d
KC823054	2012	Anhui	PCV2d	PP927800	2024	Anhui	PCV2d
KC823053	2012	Jiangsu	PCV2b	PP927801	2024	Shandong	PCV2a
KC823055	2012	Jiangsu	PCV2d	PP927802	2024	Shandong	PCV2d
KC823056	2012	Anhui	PCV2d	PP927803	2024	Shandong	PCV2d
KC823057	2012	Jiangsu	PCV2d	PP927804	2024	Jiangsu	PCV2d
KX668490	2015	Jiangsu	PCV2d	PP927805	2024	Jiangsu	PCV2d
KX668491	2015	Jiangsu	PCV2d	PP927806	2024	Anhui	PCV2d
KX668492	2015	Anhui	PCV2d	PP927807	2024	Jiangsu	PCV2d
KX865092	2015	Anhui	PCV2b	PP927808	2024	Anhui	PCV2d
KX668489	2016	Jiangsu	PCV2d	PP927809	2024	Shandong	PCV2b
KX865093	2016	Jiangsu	PCV2b	PP927810	2024	Anhui	PCV2b
KX865094	2016	Jiangsu	PCV2d	PP927811	2024	Anhui	PCV2b
KY659550	2016	Jiangsu	PCV2a	PP927812	2024	Anhui	PCV2b
KY659551	2016	Anhui	PCV2b	PP927813	2024	Shandong	PCV2d
KY659552	2016	Anhui	PCV2d	PP927814	2024	Shandong	PCV2d
KY659553	2016	Jiangsu	PCV2d	PP927815	2024	Anhui	PCV2a
KY659554	2016	Anhui	PCV2d	PP927816	2024	Anhui	PCV2b
				PP927817	2024	Anhui	PCV2d
				PP927818	2024	Shandong	PCV3b
				PP927819	2024	Anhui	PCV3a-2
				PP927820	2024	Anhui	PCV3b
				PP927821	2024	Shandong	PCV3b
				PP927822	2024	Shandong	PCV3b
				PP927823	2024	Shandong	PCV3b
				PP927824	2024	Anhui	PCV3b
				PP927825	2024	Anhui	PCV3a-2
				PP927826	2024	Anhui	PCV3a-2
				PP927827	2024	Shandong	PCV3b

Table S5. The information on 31 referenced PCV2 strains and 36 referenced PCV3 strains.

GenBank Accession no.	Genotype	Sequence name	Country
HM038034.1	PCV2a	Porcine circovirus 2 strain LG complete genome	Heilongjiang, China
AF055392.1	PCV2a	Porcine circovirus 2 from Canada complete genome(Strain 1010)	Canada
EU503040.1	PCV2b	Porcine circovirus 2 strain YangZhou0705 complete genome	Jiangsu, China
HM641752.1	PCV2b	Porcine circovirus 2 strain DBN-SX07-2 complete genome	Beijing, China
AY686764.1	PCV2b	Porcine circovirus 2 strain ZJ complete genome	Jiangsu, China
AY181947.1	PCV2d	Porcine circovirus type 2 strain SD complete genome	Shandong, China
MF616426.1	PCV2a	Porcine circovirus 2 isolate URUF21, complete genome	Uruguay
MF616425.1	PCV2a	Porcine circovirus 2 isolate URU8, complete genome	Uruguay
MF142266.1	PCV2a	Porcine circovirus 2 isolate S50-1 replicase and capsid protein genes, complete cds	Hunan, China
AY391729.1	PCV2b	Porcine circovirus type 2 strain NB0301, complete genome	Zhejiang, China
AY536756.1	PCV2b	Porcine circovirus 2 strain HuZhou0301, complete genome	Zhejiang, China
AY578327.1	PCV2b	Porcine circovirus 2 strain JS2003 from China, complete genome	Zhejiang, China
EU148504.1	PCV2c	Porcine circovirus 2 isolate DK1987PMWSfree, complete genome	Denmark
EU148505.1	PCV2c	Porcine circovirus 2 isolate DK1990PMWSfree, complete genome	Denmark
EU148503.1	PCV2c	Porcine circovirus 2 isolate DK1980PMWSfree, complete genome	Denmark
MF616427.1	PCV2d	Porcine circovirus 2 isolate URU87, complete genome	Uruguay
KU041852.1	PCV2d	Porcine circovirus 2 isolate JL-Z-CHN-2014-PCV2-PCV2d, complete genome	Jilin, China
KU041856.1	PCV2d	Porcine circovirus 2 isolate SY-L-3-CHN-2015-PCV2-PCV2d, complete genome	Jilin, China
MF589524.1	PCV2e	Porcine circovirus 2 isolate PCV2-CN/FuJian-625-2017, complete genome	Fujian, China

MF589523.1	PCV2e	Porcine circovirus 2 isolate PCV2-CN/FuJian-612-2017, complete genome	Fujian, China
KT795290.1	PCV2e	Porcine circovirus 2 strain USA/45358/2015, complete genome	America
KT369070.1	PCV2f	Porcine circovirus 2 strain Papuan 17.1, complete genome	Australia
KT369069.1	PCV2f	Porcine circovirus 2 strain Papuan 08.1, complete genome	Australia
KT369068.1	PCV2f	Porcine circovirus 2 strain Papuan 05.1, complete genome	Australia
AY713470.1	PCV2g	Porcine circovirus 2, complete genome	Germany
EU547457.1	PCV2g	Porcine circovirus 2 isolate GS02 replicase protein gene, complete cds	Gansu,China
EU547460.1	PCV2g	Porcine circovirus 2 isolate GS12 replicase protein gene, complete cds	Gansu,China
EU547459.1	PCV2g	Porcine circovirus 2 isolate GS11 replicase protein gene, complete cds	Gansu,China
KT336603.1	PCV2h	Porcine circovirus 2 isolate NN4, complete genome	Vietnam
KT336602.1	PCV2h	Porcine circovirus 2 isolate PT6, complete genome	Vietnam
KY354038.1	PCV3a-1	Porcine circovirus 3 strain CN/Hubei-610/2016, complete genome	Hubei, China
KY354039.1	PCV3a-1	Porcine circovirus 3 strain CN/Hubei-618/2016, complete genome	Hubei, China
KX778720.1	PCV3a-1	Porcine circovirus 3 strain PCV3-US/MO2015, complete genome	America
KY996337.1	PCV3a-1	Porcine circovirus 3 isolate PCV3/KU-1601, complete genome	South Korea
KY865242.1	PCV3a-1	Porcine circovirus 3 isolate CHN_Shanghai_0706_2016, complete genome	Shanghai, China
KY075993.1	PCV3a-1	Porcine circovirus 3 strain PCV3/CN/Chongqing-155/2016, complete genome	Chongqing, China
KY996340.1	PCV3a-2	Porcine circovirus 3 isolate PCV3/KU-1604, complete genome	South Korea
MF611876.1	PCV3a-2	Porcine circovirus 3 strain PCK3-1701, complete genome	South Korea
MF079253.1	PCV3a-2	Porcine circovirus 3 strain PCV3-BR/RS/6, complete genome	Brazil
KY075986.1	PCV3a-2	Porcine circovirus 3 strain PCV3/CN/Fujian-5/2016, complete genome	Fujian, China
MF611877.1	PCV3a-2	Porcine circovirus 3 strain PCK3-1702, complete genome	South Korea
KY075987.1	PCV3a-2	Porcine circovirus 3 strain PCV3/CN/Fujian-12/2016, complete genome	Fujian, China

KY075988.1	PCV3a-2	Porcine circovirus 3 strain PCV3/CN/Henan-13/2016, complete genome	Henan, China
MF155643.1	PCV3a-IM	Porcine circovirus 3 strain PCV3-China/GX2016-3, complete genome	Guangxi, China
MF155642.1	PCV3a-IM	Porcine circovirus 3 strain PCV3-Chian/GX2016-2, complete genome	Guangxi, China
MF155641.1	PCV3a-IM	Porcine circovirus 3 strain PCV3-China/GX2016-1, complete genome	Guangxi, China
MF589102.1	PCV3a-IM	Porcine circovirus 3 strain PCV3/CN/Guangdong-HY1/2016, complete genome	Guangdong, China
KX898030.1	PCV3a-IM	Porcine circovirus 3 strain PCV3-US/MN2016, complete genome	America
KY865243.1	PCV3b	Porcine circovirus 3 isolate CHN_Shanghai_0708_2016, complete genome	Shanghai, China
KY075991.1	PCV3b	Porcine circovirus 3 strain PCV3/CN/Chongqing-148/2016, complete genome	Chongqing, China
KY075989.1	PCV3b	Porcine circovirus 3 strain PCV3/CN/Jiangxi-62/2016, complete genome	Jiangxi, China
MF079254.1	PCV3b	Porcine circovirus 3 strain PCV3-BR/RS/8, complete genome	Brazil
MF162298.1	PCV3b	Porcine circovirus 3 isolate PCV3-IT/CO2017, complete genome	Italy
KY996343.1	PCV3b	Porcine circovirus 3 isolate PCV3/KU-1607, complete genome	South Korea
MF162299.1	PCV3b	Porcine circovirus 3 isolate PCV3-IT/MN2017, complete genome	Italy
KY421348.1	PCV3b	Porcine circovirus 3 isolate PCV3-CHN/CC2016, complete genome	Guangdong, China
KY996342.1	PCV3b	Porcine circovirus 3 isolate PCV3/KU-1606, complete genome	South Korea
MF589105.1	PCV3b	Porcine circovirus 3 strain PCV3/CN/Guangdong-SG1/2016, complete genome	Guangdong, China
MF589104.1	PCV3b	Porcine circovirus 3 strain PCV3/CN/Guangdong-MX3/2015, complete genome	Guangdong, China
MF589106.1	PCV3b	Porcine circovirus 3 strain PCV3/CN/Jiangxi-3/2016, complete genome	Jiangxi, China
MF405271.1	PCV3b	Porcine circovirus 3 isolate PCV3/CN/GDSJ1/2017, complete genome	Guangdong, China
MF405274.1	PCV3b	Porcine circovirus 3 isolate PCV3/CN/GXLJ2/2017, complete genome	Guangxi, China
MF069115.1	PCV3b	Porcine circovirus 3 isolate PCV3/CN/GDLC1/2016, complete genome	Guangdong, China
MF405272.1	PCV3b	Porcine circovirus 3 isolate PCV3/CN/GDBL1/2017, complete genome	Guangdong, China

MF405277.1	PCV3b	Porcine circovirus 3 isolate PCV3/CN/GXHJ2/2017, complete genome	Guangxi, China
